# Women’s Unique Health Needs and the Sustainable Development Goals: A Meeting Report from the 79th United Nations General Assembly Science Summit

**DOI:** 10.1177/15409996251377432

**Published:** 2025-11-10

**Authors:** Irene O. Aninye, Sarah Chew, Nicole Althaus, Michele Barry, Ru-Fong Cheng, Carolyn Harris, Eleanor Nwadinobi, Beth Battaglino, Aisha K. Brooks, Janna Guinen, Anuradha Gupta, Mitzi Krockover, Rachel Sturke, Marleen Temmerman

**Affiliations:** ^1^ Society for Women’s Health Research, Washington, District of Columbia, USA.; ^2^ ECHAlliance, Dublin, Ireland.; ^3^ Center for Innovation in Global Health, Stanford University, Stanford, California, USA.; ^4^ Gates Foundation, Seattle, Washington, USA.; ^5^ Parliament of the United Kingdom, London, England.; ^6^ Medical Women’s International Association, Geneva, Switzerland.; ^7^ HealthyWomen, Middletown, New Jersey, USA.; ^8^ United States Public Health Service, Washington, District of Columbia, USA.; ^9^ HLTH Foundation, New York, New York, USA.; ^10^ Global Immunization, Sabin Vaccine Institute, Washington, District of Columbia, USA.; ^11^ Woman Centered, LLC, Scottsdale, Arizona, USA.; ^12^ Fogarty International Center, Bethesda, Maryland, USA.; ^13^ Centre of Excellence Women and Child Health, Aga Khan University, Nairobi, Kenya.

**Keywords:** global health, health care policy, research and development, sustainable development goals, technology innovation, workforce development

## Abstract

Biological sex, hormones, and gender uniquely affect health and disease, often resulting in disparities for women across the lifespan and from different racial and ethnic groups, geographical locations, and socioeconomic backgrounds. Without intentional investment and infrastructure to support good health and wellbeing for women, half of the world’s population remains vulnerable to preventable morbidity and mortality. The Society for Women’s Health Research and ECHAlliance–The Global Health Connector convened a women’s health program as part of the 10th Annual Science Summit during the United Nations 79th General Assembly. This hybrid event was held in New York, NY, (USA) and virtually in September 2024. The program entitled “Women’s Unique Health Needs and the Sustainable Development Goals” comprised a series of discussions about preventive care, policy infrastructure, and workforce inclusivity to promote advancements in women’s health care, research, leadership, and policymaking to achieve the Sustainable Development Goals. This report highlights key points from each session and opportunities to engage in efforts to close the global gender health gap.

## Introduction

Biological sex, hormones, and gender uniquely, differently, and disproportionately affect health and disease, often resulting in disparities for women across the lifespan.^1^ These challenges are further exacerbated in women from racial and ethnic minority populations, low- and middle-income countries (LMICs), and under-resourced geographic locations. The unique needs and critical role of women in the global health care ecosystem are underrepresented and undervalued, resulting in persistent gaps in awareness and investment in women’s health research. A comprehensive strategy to advance research on women’s health must be developed and implemented to address this deficiency for half of the world’s population.

The Society for Women’s Health Research (SWHR) and ECHAlliance–The Global Health Connector were invited to convene a women’s health event as part of the 10th Annual Science Summit during the United Nations 79th General Assembly. The program, “Women’s Unique Health Needs and the Sustainable Development Goals,” was a hybrid event held in person in New York, NY, (USA) and virtually on September 24, 2024, comprising a series of presentations, panels, and roundtables to discuss the state of women’s health care delivery, research innovation, and policy implications across diverse global contexts. Leaders across continents and with expertise in women’s health, medicine, and government gathered to identify opportunities to address this grand challenge, with a focus on preventive care, policy infrastructure, and workforce inclusivity to advance women’s health along the pursuit of the Sustainable Development Goals (SDGs). This report highlights key points from each session and opportunities for women’s health stakeholders to engage in efforts to close the global gender health gap. The speakers for the program are listed in Table 1.

**Table 1.** UNGA79 Women’s Health Event Speakers and Moderators

Speakers	Title and department	Institution affiliation
Mala Adiga, JD, MPH	Deputy Assistant to the President, Policy Director to the First Lady	United States White House
Michele Barry, MD, FACP	Director for Center of Innovation in Global Health	Stanford University
Beth Battaglino, RN-C	CEO	HealthyWomen
Aisha K. Brooks, DNP, MPH, RN, FAAN	Senior Health Policy Administrator	United States Public Health Service
Ru-fong Cheng, MD, FACOG	Director, Women’s Health Innovations	Gates Foundation
Janine Austin Clayton, MD, FARVO	Director, Office of Research on Women’s Health	National Institutes of Health (USA)
Jess Guenzl	Principal	137 Ventures
Janna Guinen	Executive Director	HLTH Foundation
Anuradha Gupta	President, Global Immunization	Sabin Vaccine Institute
Carolyn Harris	Labor MP for Neath and Swansea East	Parliament of the United Kingdom
Mitzi Krockover, MD	Founder and CEO	WomenCentered, LLC
Eleanor Nwadinobi, MBBS, EMA, FAAC	President	Medical Women’s International Association
Rachel Sturke, PhD, MPH, MIA	Acting Deputy Director, Fogarty International Center	National Institutes of Health (USA)
Marleen Temmerman, MBS, MD, MPH, PhD	Center of Excellence Women and Child Health	Aga Khan University

Moderators/Discussants	Title and department	Institution affiliation
Nicole Althaus	U.S. Ambassador	ECHAlliance
Irene O. Aninye, PhD	Chief Science Officer	Society for Women’s Health Research
Brian O’Connor	Chair	ECHAlliance
Magdalena Skipper, PhD	Editor in Chief	Springer Nature (United Kingdom)

## Opening Remarks

*Presenters: Brian O’Connor (ECHAlliance); Irene O. Aninye, PhD (Society for Women’s Health Research)*

Mr. O’Connor and Dr. Aninye welcomed the in-person and online audience to this Global Health Connector Event at the Cure venue and outlined the goals of utilizing this global platform to better understand sex and gender differences in health and disease and to improve societal health and wellness outcomes by addressing gaps and unmet needs in women’s health. While gynecological and maternal health are important topics, women’s health is much broader and must address all health conditions, life stages, and diseases that present disparities in women, either through incidence or through outcomes.

In 2021, an economic and societal impact analysis conducted by the Women’s Health Access Matters organization showed that investing $300 million in health research focused on women across three specific disease areas would generate over $13 billion in economic returns through improved quality of life, reduced health care costs, and more productive years added back to the workforce.^2^ Such returns on investment could multiply exponentially if investment was expanded to the full spectrum of health topics that disproportionately or uniquely affect women and if these actions were not just engaged in the United States but on all seven continents.

The event’s program was designed with the aim to connect people and organizations; discuss not just problems, but solutions; amplify awareness to prevent repeating past mistakes and reinventing previous models; and accelerate efforts by translating aspirations into action with sound methodology and funding support. A historical snapshot of women in medical research over the last 50 years in the United States further underscored the importance of scientific experts and policymakers working together to address gender health gaps across the world.

Lastly, attendees were challenged to consider how the discussions of the day influence and are influenced by multiple SDGs beyond good health and wellness (Goal 3). Other goals offered for consideration included gender equality (Goal 5) and partnerships for the goals (Goal 17). For example, clean water and sanitation (Goal 6) are crucial for promoting menstrual health in women and girls; innovation and infrastructure (Goal 9) support growth and accountability for the femtech industry; and reduced inequalities (Goal 10) ensure that women across all nations can access and engage in medical advances and quality care when needed.

SWHR and ECHAlliance offered their partnership as an example of a collaboration that is elevating the conversation and implementing action to advance women’s health.

## A Call to Action: Women’s Unique Health Needs and the SDGs

*Presenter: Ru-fong Cheng, MD, FACOG (Gates Foundation); Moderator: Irene O. Aninye, PhD (Society for Women’s Health Research)*

Dr. Cheng posited that there are countless benefits to everyone when we invest in women’s health. An investment in women’s health yields exponential returns because when women are well, they do well; however, a lack of data persists in women’s health. There is a gender gap in medical research, with less than half (42%) of full-time faculty in United States institutions and 18% of department chairs being women.^3^ Moreover, only 2% of top pharmaceutical companies are led by women. Women are also underrepresented in clinical trials, critically hindering our ability to eliminate the disparities women experience in conditions like cardiovascular disease, cancers, and psychiatric illnesses. Women are seven times more likely to be misdiagnosed than men and spend 25% more of their lives in poor health compared with men.^4^ These adverse health outcomes are even worse for women of color and women living in LMICs.

Women’s health has implications across the SDGs, with repercussions in work life and the global economy. In alignment with SDG Goal 3 (good health), when women are healthy, they are more enabled to keep their families healthy as well. Promoting quality education (Goal 4) for women and girls positions them to better understand their health and have shown to improve family planning, which, in turn, is supported by gender equality (Goal 5) and bodily autonomy.^5^ Women are likely to stop family planning methods when they do not match or fit their lifestyles, or they are too expensive, mandating a need to better respond to women’s lives and preferences when developing sustainable solutions. Investing in women’s health has the ability to increase the global economy (Goal 8) by $1 trillion, translating to a 1.7% increase in GDP annually per capita.^4^

Innovations and adherence to interventions must be accessible and meet the needs, preferences, and lifestyles of women and girls. A key recommendation to eliminate barriers to accessing care, particularly in LMICs, was prioritizing simple, low-cost point-of-care diagnostics. Dr. Cheng issued a call to action across the health care ecosystem:

• Researchers must be curious, ask questions, and challenge the status quo of current health care resources and practices;
• Government leadership should play a central role in instituting policies that strengthen the research pipeline and ensure accessibility; and
• Private sector partners and funders are needed to support innovation and advance priorities through resources and financial investments.

By working across sectors, Dr. Cheng is confident that we can build a better women’s health ecosystem.

## White House Initiative on Women’s Health Research

*Presenter: Mala Adiga, JD, MPH (United States White House)*

On March 18, 2024, the President of the United States, Joe Biden, signed an Executive Order on Advancing Women’s Health Research and Innovation (EO14120). Adiga joined the proceedings to give special remarks and outline progress to date made toward this federal call to action. Agency commitments to this first-ever White House initiative included $100 million from the Advanced Research Projects Agency for Health for a Sprint for Women’s Health initiative, a $200 million increased investment by the National Institutes of Health (NIH) to close gaps in women’s health research across the lifespan, and a $500 million pledge from the Department of Defense dedicated to women’s health in its research portfolio. These investments reflected crucial momentum in funding women’s health research across the United States.

Adiga also emphasized the importance of interdisciplinary and cross-sector collaborations to address women’s health disparities in the United States and internationally. While the session highlighted investments in the United States, it also reflected broader changes that are needed to fundamentally shift the approach to funding women’s health research everywhere.

## Transitioning Women’s Health from Treatment to Prevention

*Panelists: Aisha K. Brooks, DNP, MPH, RN, FAAN (U.S. Public Health Service), Anuradha Gupta (Sabin Vaccine Institute); and Rachel Sturke, PhD, MPH, MIA (Fogarty International Center); Moderator: Nicole Althaus (ECHAlliance)*

As of 2021, 4.5 billion people globally were not covered by essential health services, which include preventive care.^6^ During this panel session, speakers explored how health care leaders—both domestic and global—can strengthen their countries’ approaches to preventive care, particularly for diseases that disproportionately affect women. The panel used access to preventive HPV vaccines and cervical cancer screening as a model of impactful preventive care—that if systemically implemented in LMICs over a 10-year period, would avert 5.2 million cervical cancer cases and 3.7 million deaths.^7^ The panel discussion highlighted key intervention strategies for health care systems aiming to expand prevention for their citizens, including capacity-building, comprehensive health care integration, and recognizing lived experiences.

### Capacity building

Health care leaders should prioritize the development of infrastructure and partnerships that can facilitate the delivery of preventive care services across multiple levels—national policy, organizational, and community. Building this capacity requires more than technology and partnerships; it also requires strategic alignment around access, care coordination, and cultural values. Public-private partnerships and digital tools can play a vital role in addition to policies that reduce financial barriers to screening, reflect the caregiving roles many women hold, and drive care models that are rooted in compassion and accountability. These strategies are essential to bridging the divide between treatment and prevention, especially for women and young girls.

### Integration of preventive health care

Health systems must identify strategies to integrate primary and secondary interventions to eliminate harmful health care silos. Preventive care must function within systems that enable seamless access, thus reducing the burden on individual women to navigate fragmented services. This includes ensuring that services are routinely evaluated to determine whether they are meeting the needs of the populations they are intended to serve, and integration efforts must be guided by outcomes, not assumptions. Such synergies between disease prevention and health promotion should also be bolstered by collaborations between health care systems and community-based health organizations.

### Understanding the lived experiences of women

Advancing preventive care requires not only access to innovative and effective medicines (such as vaccines) but also a deep understanding of the lived experience of the intended populations of women. This understanding should encompass the upstream structural factors that shape health, such as housing, financial security, caregiving responsibilities, and transportation. These conditions often hinder access to preventive care and account for 45–60% of variations in health status and outcomes between populations.^8^ A nuanced understanding of these realities is essential for designing systems and policies that are both responsive and sustainable.

The consensus recommendation of the panel was that any shift toward prevention must adopt a holistic approach that considers individual women and the broader community. Transitioning from a treatment-centered to a prevention-focused model requires comprehensive, multi-level policy changes. Equally important is the meaningful inclusion of women in these processes, as their perspectives shape relevant, effective, and widely accepted health policies.

## Ensuring Accountability: Women in Research, Health Care, and Policy Leadership

*Presenters: Janine Austin Clayton, MD, FARVO (U.S. National Institutes of Health), and Carolyn Harris (Parliament of the United Kingdom); Moderator: Magdalena Skipper, PhD (Springer Nature, United Kingdom)*

Dr. Skipper opened the session by asking the audience to reflect on accountability systems and actors for public health and safety interventions in their local contexts. Dr. Skipper highlighted well-known gender gaps in design and technology innovations, such as the frequent use of male crash test dummies to collect data on safety standards and engineering applications or the standard temperature settings in office buildings being calibrated based on male internal body temperatures.^9,10^

In her presentation, Dr. Clayton discussed how academic research should take accountability for gaps in women’s health and sex differences research. Collaborations and credit for women in research is systemically limited—women are significantly less likely to publish first-author articles or be recognized for their research. Dr. Clayton outlined strategies, policies, practices, and programs that the Office of Research on Women’s Health at the National Institutes of Health (USA) uses to promote accountability throughout the biomedical research enterprise. As the architect of the NIH’s Policy on Sex as a Biological Variable (SABV) that was introduced in 2016, Clayton explained how unidimensional research that overly relies on male subjects in preclinical studies, clinical studies that center a 70-kg male model, and lack of attention to diseases and conditions of women have created significant biomedical knowledge gaps. Disaggregating results by sex can often reveal stark differences that were “hiding in plain sight.”^11^

Highlighted NIH programs that elevate women’s health research included Specialized Centers of Research Excellence on Sex Differences^12^; Bridging Women of Understudied, Underrepresented, and Underreported (U3) Populations into Focus administrative grant supplements; and a web-based resource library and learning courses to support scientists as they address these gender gaps in research design and implementation. Dr. Clayton wove data throughout her presentation to support the Institutes’ efforts, serving as the ultimate example that inclusion of SABV in research enhances rigor and accountability for addressing gender health gaps and improving outcomes for all individuals.

MP Harris then walked attendees through her journey of successfully championing menopause health care and employment rights for women in the United Kingdom. She used her own range of menopause symptoms—which included hot flushes, vaginal dryness, and mood disturbances—as conversation starters to disrupt the system and challenge her policymaking peers to talk openly about women’s health issues. She used grassroots efforts to increase political attention among male and female members of Parliament, holding them accountable for policies that were not sufficiently addressing the needs of more than half of their constituents. Harris recognized that mandatory workplace policies surrounding menopause challenges for women were not feasible due to complex government infrastructure and bureaucracy. Moreover, a singular policy is not likely to be able to address the breadth of needs for all women, given the vast differences in their experiences, industries, and geographical jurisdictions.

Harris’ strategy involved forming an all-party parliamentary group on menopause to convince the government to rethink the way the country and its leaders thought about menopause. The resolve was to address prescription policies that would increase access to menopause hormone therapy drugs for women in England who had to pay, fees unlike their counterparts in Wales and Northern Ireland. Additional efforts focused on destigmatizing menopause in the public and workplaces. Now, the United Kingdom is a recognized leader driving workplace equity and solutions surrounding menopause.

The approaches presented during this session to strengthen national infrastructures that advance women’s health provide tools that can be applied in a wide variety of contexts across the globe. Policy solutions that are inclusive of the unique needs of women must be both flexible and applicable across diverse settings and situations to ensure maximum uptake and adherence. Financial and programmatic resources must be dedicated at a national level and filtered down to local contexts so that they can be accessed by individuals to directly meet their needs—whether a scientist is requesting additional research funds or a woman is filling prescription medications. Finally, it is imperative that trustworthy messengers are engaged to champion efforts, build awareness, facilitate education, and reduce stigma associated with long-ignored women’s health issues.

## Perspectives from Women Leaders in Women’s Health

*Panelists: Janine Austin Clayton, MD, FARVO (National Institutes of Health, USA); Michele Barry, MD, FACP (Stanford University); and Eleanor Nwadinobi, MBBS, EMA, FAAC (Medical Women’s International Association; Moderator: Irene O. Aninye, PhD (Society for Women’s Health Research)*

This session began with speakers sharing their personal journeys to leadership and offering insights into what it means to promote women’s health across their respective fields. Despite women comprising almost 70% of the global health care workforce,^13^ several key challenges to achieving gender parity in health leadership were highlighted, including systemic barriers to women advancing within male-dominated institutional hierarchies; tensions between career aspirations and family planning goals; and health and safety concerns unique to women and girls in the work environment.

Each panelist proposed strategies to address these challenges, drawing from their individual experiences as women leaders. Strategies emphasized the crucial role of women in health care leadership, particularly in guiding other women as they navigate higher education and work spaces in Science, Technology, Engineering, Mathematics (STEM), where women comprise only 28.2% of the global workforce.^14^ Speakers addressed the need for both mentors and sponsors to provide support and opportunities for women, particularly based on their personal experiences of success over the obstacles they encountered. The discussion underscored the need for institutions to develop and promote opportunities that allow women to balance career and family, reframing these as complementary, not opposing, forces. With one out of four women experiencing sexual assault while doing fieldwork,^15^ the panel called for the establishment of codes of conduct and safeguarding policies by leaders—both male and female—to address issues of physical and emotional safety and violence in the workplace.

In closing, the panelists reiterated the importance of collaboration, communication, and championing women leaders, emphasizing the importance of a collective effort to advance gender parity in global health leadership.

## Strengthening the Maternal Health Workforce

*Panelists: Beth Battaglino, RN-C (HealthyWomen); Eleanor Nwadinobi, MBBS, EMA, FAAC (Medical Women’s International Association); and Marleen Temmerman, MD, MPH, PhD (Aga Khan University); Moderator: Irene O. Aninye, PhD (Society for Women’s Health Research)*

The global maternal mortality ratio decreased by 34% from 2000 to 2020, yet unacceptable numbers of pregnancy-related deaths are still occurring worldwide.^16^ Successfully achieving the SDGs means addressing domestic and international disparities, especially for areas and interventions that have demonstrated their effectiveness. This closed roundtable, held as a concurrent session after the main program, highlighted strategies to improve maternal health outcomes through leveraging care by a diverse set of skilled health professionals—from pre-pregnancy to postpartum.

While obstetric-gynecological providers are well-known maternal health professionals, there is often a local need or personal preference for the expertise of midwives, doulas, and community health workers to support women in geographically diverse and emergency settings. A diverse workforce is also essential, as sex and gender concordance and racial/ethnic alignment have also demonstrated impacts on patient-provider interactions regarding compassion, trust, communication, transparency, and respect. A key lesson learned during the COVID-19 pandemic was how to be flexible and adapt processes to provide essential care when existing infrastructure is compromised or lacking. With an estimated global shortage of 5.9 million nurses, midwives have been projected to support up to 90% of this global need for reproductive, maternal, newborn, and adolescent health interventions.^17^ Currently, they represent less than 10% of the maternal health workforce.

Attendees discussed disturbing trends in violence against women that can have physical, psychological, and emotional consequences—both short and long term. Up to 6% of women aged 15 years or older experience non-partner sexual violence, and the impacts extend into adverse pregnancy outcomes.^18^ An estimated 41% of affected women are more likely to have preterm births and 16% are more likely to experience miscarriages. Violence against women is very preventable if the necessary health and policy stakeholders provide the comprehensive health care, referrals, and infrastructure to support services that at-risk and affected women need to address these challenges. Safeguarding is also not just for the patients; the individuals working to save lives need their own environments and lives protected. As shortages and barriers in the maternal health care and workforce spaces are addressed, considerations must include who and where deployment is most needed to improve unsafe working environments and the aforementioned negative outcomes.

## Role of Femtech in the Women’s Health Care Ecosystem

*Panelists: Jess Guenzl (137 Ventures); Janna Guinen (HLTH Foundation); and Mitzi Krockover, MD (WomenCentered, LLC); Moderator: Nicole Althaus (ECHAlliance)*

A second closed roundtable session was held to convene an intimate group of key stakeholders and leaders in the femtech and venture capital space. This roundtable examined the rise in technological innovations to address women’s health issues—often referred to as femtech (female technology)—which has seen a 1,000% increase in businesses in the past decade.^19^ Panelists described emerging opportunities within the health tech ecosystem and the need for diverse stakeholders to drive innovation specifically for women’s health and women founders. Only 2% of total venture funding for health innovators went to women’s health, totaling $481 million invested in women’s health companies in 2023.^19^ From menstrual products, fertility solutions, gynecological devices, and menopause symptom management to diagnostics and therapies for Alzheimer’s disease, autoimmune disorders, and cardiovascular disease, there is untapped potential for women’s health innovations to enhance the global economy by up to $1 trillion by 2040.^4^

The femtech ecosystem is robust, including diverse communities with needs that span social identities and women across the lifespan; science and data professionals that drive and support solutions; entrepreneurs to convert science into innovation; funders to endorse public and private research and innovation; and regulators to manage rollouts, application, and accessibility. While progress has been made to support more early-stage female investors, women founders, and funds focused on women’s health innovations, gender bias still exists in femtech, particularly in late-stage investing. In 2020, men made almost 90% of the investment decisions in the United States’ venture firms, and innovations with male teams were more likely to receive funding.^19^ The session stressed the need to address this gender bias in health tech founding, funding, data collection, and solutions to improve women’s health outcomes.

Attendees also discussed implementing femtech solutions across diverse economic settings, as well as regulatory policies and practices that would support and not hinder the pace of innovation. The roundtable closed with prompts to further consider strategies that promote digital health equity and encourage male allyship in the femtech industry.

## Closing Remarks

*Presenter: Irene O. Aninye, PhD (Society for Women’s Health Research)*

Throughout the day, speakers echoed the need for increased funding for research and innovation, personal stories to humanize statistics, and additional education about women’s health research and care.

The program closed by revisiting the charge for the day and Dr. Aninye encouraging every attendee to complete a simple “1-2-3” action before leaving:

1. Name at least one immediate action you can take to address women’s unique health needs within your sphere of influence.
2. Identify two resources or organizations that you can include on your go-to list for reliable information to assist your efforts.
3. Write down at least three potential partners who you will reach out to for collaboration, preferably within the next 60 days.

When every individual identifies a personal investment in women’s health and commits to making a change to support women’s health across budgets and labs benches, the gender health gap will finally begin to close.
